# Supplementary material for: Facets of individual-specific health signatures determined from longitudinal plasma proteome profiling
Source: eBioMedicine. 2020 Jul 3;57:102854. doi: 10.1016/j.ebiom.2020.102854 (PMC7334812; doi:10.1016/j.ebiom.2020.102854)
Supplement: Supplementary file 1 [file mmc1.docx]

Supplementary Information for

**Facets of individual-specific health signatures determined from longitudinal plasma proteome profiling**

Tea Dodig-Crnković, Mun-Gwan Hong, Cecilia Engel Thomas, Ragna S. Häussler, Annika Bendes, Matilda Dale, Fredrik Edfors, Björn Forsström, Patrik K.E. Magnusson, Ina Schuppe Koistinen, Jacob Odeberg, Linn Fagerberg, Anders Gummesson, Göran Bergström, Mathias Uhlén and Jochen M. Schwenk^*^

* Corresponding author **Address:** Science for Life Laboratory, Department of Protein Science, KTH-Royal Institute of Technology, Tomtebodavägen 23, 171 65 Stockholm, Sweden. **Email**: jochen.schwenk@scilifelab.se

Supplementary Information

**Description of experimental design**

In the S3WP study, we have analysed EDTA plasma from 101 individuals collected from the four first visits in the S3WP study. Visit five and six were later included for validation of a subset of protein targets related to seasonal variation. Following each completed visit, plasma samples were randomized within an assay and analysed together with all previously collected visits (Fig. 1). Each assay consisted of samples distributed across 96-well microtiter plates, balanced with respect to age and sex. Every plate contained at least four wells for technical quality control: one well with buffer (negative control) and triplicates of pooled plasma for the assessment of inter- and intra-plate variability. Batch effects were evaluated by repeating complete assay runs. The fourth assay, which consisted of samples collected from all four visits, included the restriction that all samples from an individual were randomized within the same microtiter plate. In addition, one 96-well microtiter plate was analysed twice for the assessment of batch effects. To minimize the risk of protein degradation caused by repeated freezing and thawing cycles, the fourth assay included sample aliquots that had only been thawed once previously.

Out of 101 individuals, 99 participants completed all four visits. One individual was excluded from the analysis due to a sample swap during visit three sample shipment. Five individuals had a sample from one visit that failed the sample preparation step and therefore the participants were completely excluded from the data set. In total, 93 individuals with one sample from each visit were included in the downstream data analysis. One outlier (W0030) was identified across all visits (see Supplement for more details and Fig. S11). For validation analysis, 86 individuals were included that had donated samples six consecutive times in total. For GWAS, described in detail below and in Supplementary Materials and Methods, data from 101 individuals at visit 1 was used.

**Identification of IgM interference outlier**

During an initial PCA analysis, we found one subject (W0030) with consistently outlier protein profiles in the first PCA component across all four visits (Fig. S11A). Indeed, higher protein levels were measured for 11% (79/734) of all protein features (Fig. S11B). We suspected and indeed confirmed that IgM bound to several of the rabbit IgG antibodies (Fig. S11C), hence elevated IgM might have interfered with a subset of the SBA assay data. While elevated IgM levels have been mentioned as a challenge for immunoassays on the one hand,^1^ they are on the other hand frequently found in a variety of autoimmune diseases.^2^ Elevated IgM levels could even serve as an actionable clinical outcome for monoclonal gammopathy of undetermined significance, Waldenstrom's Macroglobinemia, or Multiple myeloma.^3,4^ We therefore carefully considered excluding this participant from some of the data analysis, such as for calculating proteins z-scores, because it could otherwise pick up this outlier with deviating baseline levels.

**Selecting antibodies for downstream analysis**

We created a scoring scheme that allows ranking and evaluation of each antibody in the suspension bead array (SBA) based on their reliability and performance in plasma (Supplementary Materials and Methods) (Table S3, Table S4). Antibodies with a final confidence score > 1 were chosen for further analysis (Fig. S1). The score was based on supportive data from collected correlation profiles between each antibody and repeated experiments, control beads, other antibodies directed towards the same target, and additional antibody-based technologies (sandwich assays and proximity extension assays) and antibody-free methods (mass spectrometry and GWAS annotation). For the succeeding analysis, one antibody per protein target (or protein family) was chosen by selecting the highest scoring antibody. Thereby, the scoring scheme allowed filtering from 1,450 antibodies and 33 control beads applied in the purpose of an exploratory screening array, to the final collection of 734 most reliable features.

Antibodies with reproducibility difficulties could be explained by several factors, including: (i) the intended target is too low abundant or absent, (ii) the intended target is outcompeted by other more abundant proteins with attractive epitopes, (iii) the target protein is not available due to denaturation or degradation during sample storage, thawing or freezing cycles, (iv) the epitope is masked in the native confirmation due to interactions or modifications. An antibody with high technical reproducibility may indicate that the antibody repeatably binds to the same protein or protein complexes, however, this does not always confirm that the bound protein is indeed the intended target. The applied efforts for selecting a large number of antibodies may therefore still carry a risk of off-target binding. Starting out with a high number of antibodies, each with an intended protein target but unknown degree of specificity in a plasma matrix, we set out to validate the applied binders.

We acknowledge that the scoring approach is biased to those targets that overlap between platforms, and that the comparisons do not always fully account for the complexity of antibody-antigen binding, such as co-enrichment or off-target binding events. As an example, two proteomics methods may both bind the correct protein but still not correlate with each other if they capture the protein of interest to different degrees, or if the methods measure different versions (proteoforms) of the same protein. The validation process can be time-consuming and end in conflicting results between methods, however, it is necessary to strive for high quality binders in affinity-based assays before taking any findings further for biological interpretation.

**Seasonal variation**

To capture possible seasonal effects induced by changes in temperature, sunlight exposure, lifestyle or other annual environmental factors occurring in Sweden, we applied a linear regression model that included trigonometric functions of each protein profile. Among all proteins, we observed only a few significant associations (Fig. S5A). There were two antibodies targeting partially overlapping regions on filamin-A (FLNA) that revealed seasonal fluctuation (Fig. S5B-C) (FDR P = 1·11×10^-6^ and P = 1·88×10^-6^). In addition, plasma profiles of GRB2-related adapter protein 2 (GRAP2) and biliverdin reductase B (BLVRB) (Fig. S5D) also changed in a seasonal manner (FDR P = 6·40×10^-3^ and P= 8·78×10^-3^). Inter-assay and inter-visit plots illustrating the technical and biological variation of FLNA and BLVRB are in Fig. S5E-G. To support these observations, we analysed two additional longitudinal samples (visit 5 and 6) from the study participants, which confirmed the seasonal variation of FLNA (FDR P = 1·42×10^-5^ and P = 1·10×10^-2^) and BLVRB (FDR P = 2·99×10^-4^), but not GRAP2.

FLNA is a cytoskeletal protein that can leak into the blood while otherwise promoting the branching of the actin filaments and linking these to membrane glycoproteins on a cellular level.^5^ BLVRB is an enzyme leaking from hematopoietic cells and the liver. It acts in the heme metabolism^6^ and was described as a predictive biomarker for cardiovascular events.^7^ Observing seasonality for BLVRB was consistent with a comprehensive study of mRNAs,^8^ however, the physiological reason for fluctuating FLNA and BLVRB plasma levels remain to be determined. Overall, seasonal variations had a minimal effect on the circulating proteome, but as exemplified by BLVRB, could still be a variable worth considering.

**WGCNA**

***Distribution of controls in mega modules***

WGCNA was performed for all antibodies for each visit separately to assess the distribution of controls and antibody scores in the resulting modules. Both controls and antibodies with high correlation to controls were distributed across all modules. Thus, module formation was not driven by these factors. However, an increased number of antibodies with low antibody score was observed for the green mega module. Further, module formation was not primarily driven by the measured signal intensity as evidenced by the similar signal distributions observed across all modules.

***Technical tests of mega modules and core patterns***

As multiple bead arrays sets were used (SBA1-4), we assessed whether the mega modules and patterns of the core modules derived from WGCNA on 734 proteins were driven by these array batches. For the mega modules the binder sets were relatively evenly distributed across modules, with only one module in each visit consisting primarily of proteins targeted by antibodies from SBA4. Further, the purple core pattern was entirely comprised of proteins targeted by this binder set, whereas the green core pattern was mostly comprised of binders targeted by SBA3. The other core modules consisted of proteins targeted by several different SBAs. Thus, most mega modules and core modules were not linked to a particular SBA.

The distribution of the proteins across modules was also investigated. We performed bootstrap sampling to assess the stability and consistency of the modules within each visit. For all visits the module membership of the majority of proteins remained the same across visits (Fig. S7). Thus, the module assignments obtained from WGCNA were deemed to be non-random.

**GWAS**

***Analysis for antibody annotation***

The whole genome sequences of somatic cells of the entire 101 subjects provided information on the genetic variations that individual subjects carry. Focusing on bi-allelic non-redundant SNPs, genome-wide association tests for protein profiles were performed to identify any molecular diversity driven by genetics, which likely provides high confidence of the molecular targets of the SBA assay. In total, we found 15 pQTLs (Table 1, Fig. S2). Since the affinity of the antibodies to its target protein is likely affected by the sequence of amino acids, we hypothesize that the SBA data can be linked to genetic variation that result in protein polymorphism. In other words, antibodies can bind preferentially to a particular protein variant when a missense variation introduces differences in the binding affinity rather than a difference in protein abundance. Since affinity can also be influenced by other proximal changes around the epitope, for example degree of post-translation modifications or interactions, the data from the assays may reflect not only the “primary” abundance and expression of a protein, but also “secondary” changes around the epitope identified by cis-pQTLs.

***pQTL and eQTL analysis***

For the 15 identified pQTLs, all targets have been found to be enriched for RNA expression in the liver ([http://proteinatlas.org](http://proteinatlas.org/), RNA expression overview from consensus dataset), except for CLEC3B, which is largely expressed in lung and multiple other tissues. Querying the GTEx Portal V8 ([https://gtexportal.org](https://gtexportal.org/home/)) we found nine expression quantitative loci (eQTL) matching our 14 unique pQTL hits (Table S6). For these, F12 and LRG1 had liver as the main eQTL tissue. Although leakage products from any cell or tissue type may end up in the blood stream, the most abundant plasma proteins are produced and actively secreted from the liver. eQTL hits that coincide with our pQTL analysis suggest that the abundance of circulating protein in plasma is reflecting the level of gene expression in liver. In contrast, pQTLs for which no eQTL was detected may indicate that what we measure is not proportional to gene expression, but rather to other factors that influence antibody-antigen binding such as post-translational modifications or, as discussed above, missense variation. Furthermore, we found seven splice eQTLs for which the two targets AHSG and CFH were enriched in liver.

**Supplementary Materials and Methods**

**Genome analysis S3WP study**

Genomic DNA was quantified using Qubit 2.0 Fluorometer (Invitrogen), fragmented into average 350-bp fragments using E220 focused-ultrasound sonicator (Covaris) and 1 µg of fragmented DNA was converted into sequencing ready library using TruSeq DNA PCR-free HT Sample preparation method (Illumina). The obtained library was quantified using KAPA SYBR FAST qPCR (Kapa Biosystems) and pair-end (2x150 bp) sequenced to average 30x coverage on the HiSeq X system (Illumina) using v2 flowcells. Demultiplexing was done without allowing any mismatches in the index sequences. Bioinformatic analysis of the sequence data was carried out using Mutation Identification Pipeline (version 4.0.18).^9^ Briefly, alignment was done using BWAmem using the reference genome GRCh38.p7, and single-nucleotide and insertion/deletion variants called using GATK (v 3.6). Structural variants were called using Manta (v 1.0.3). Variants in any of the 56 ACMG genes^10^ were excluded from further analysis in order to avoid secondary findings. From the filtered data of genetic variants, which were stored in variant call format, the genotypes of the 101 individuals were merged for each SNP assuming the individuals without variants on the locus had a pair of the reference allele for the SNP. After filtering for biallelic SNPs that were not rare (MAF > 5%) and for which genotyping was successful (genotype in > 90% of samples), redundant SNPs (r^2^ > 0.99 in 2 kb) were removed. Association between a protein and each of ~3.7M genetic variants was after the refinement tested using simple linear regression on allele counts with PLINK (v 1.90). P-values were Bonferroni corrected for each protein profile (0.05/3.7M). The functional classes (e.g. missense) and LD proxies of the associated SNPs were obtained from LDlink.^11^ Manhattan plots were drawn by using qqman package (v 0.1.4).

**TwinGene samples**

***Sample collection***

During 2004-2008, the Swedish Twin Registry (STR) collected blood samples from 12,614 elderly twins born between 1911-1958.^12^ A total of 22,390 twins that participated in the Screening Across the Lifespan Twin Study^13^ were invited to the TwinGene project. Participants received consent forms and health questionnaires. Subjects that enrolled in the study were sent blood sampling equipment and were instructed to contact their local healthcare facility for a health examination and sampling. Participants were asked to fast from 8 pm the day before blood was drawn. Serum was stored in liquid nitrogen and EDTA tubes of blood were used for DNA isolation with Puregene extraction kits (Gentra systems, Minneapolis, USA). Blood serum was stored at -80°C and extracted DNA at –20°C.

***Genome analysis TwinGene study***

Genomic DNA was genotyped for all available dizygotic twins and one member of each monozygotic twin pair using the Illumina OmniExpress BeadChip 700K.^12,14^ Imputation was done using the 1000 Genome reference panel (GRCh 37/hg 19, Phase 1, version 3) with Mach 1.0 and Minimac. Using PLINK (v 1.90 beta), genome-wide association study was performed on 2,592 twins. The analysis included autosomal SNPs with imputation quality (info or r^2^) higher than 0.4. To control for population stratification, the four first principal components were used as covariates in a linear regression model. In order to adjust for relatedness, the *--within* option in PLINK was applied.

**Detection of IgM**

Assays to detect the reactivity of human IgM against rabbit IgG coupled to Luminex beads was as follows: A set of 76 biotinylated samples from 19 individuals (4 visits each) was diluted 1:50 and heat treated as described for the SBA assay procedure. The samples were then added to beads carrying normal rabbit IgG (P80-102, Bethyl) to resemble HPA antibodies. After an overnight incubation, the beads were washed in 3x 100 µl PBST and incubated with 50 µl of 1 µg/ml goat anti-human-IgM-PE (#2020-09, RRID: AB_2795606, SouthernBiotech) for 60 min. Beads were detected on the FlexMap3D system (Luminex) as described for the SBA assay.

**Antibodies and creations of suspension bead arrays**

Four suspension bead array (SBA) batches comprising a total of 1,483 antibodies were created, out of which 1,450 antibodies were intended to be used for data analysis. The other 33 beads were used as controls. The control beads were without antibodies or coupled to rabbit IgG and served as negative controls, while beads conjugated to anti-human IgG or anti-albumin were used as positive controls, see Supplementary Data file S1.

Each bead has a unique ID defined by an internal fluorescent dye, which enables the identification of a bead ID within a pool of beads. In short, 1.6 µg of antibody was diluted in MES buffer to a total volume of 100 µl. Beads aliquoted in 96-well microtiter plates, with approximately half a million beads per bead ID, were washed (EL406 washer, BioTek) and the carboxylated bead surfaces were activated with N-hydroxysuccinimide (24510, Pierce, Thermo Fisher) and 1- ethyl-3(3-dimethylamino-propyl)carbodiimide (22980, Pierce, Thermo Fisher). Following 20 minutes incubation at room temperature, the beads were washed in MES buffer and the diluted antibodies were added to their assigned bead ID. After 2 h incubation, the beads were washed in PBS-T 0.05% (1xPBS (09‐9400, Medicago), 0.05% Tween20 (P9416, Sigma-Aldrich)) and the bead surfaces were blocked by the addition of Blocking Reagent for ELISA (11112589001, Roche, Sigma), supplemented with 0.1% (v/v) ProClin (48912-U, Sigma-Aldrich). SBAs were created by combining equal volumes of each bead ID. Here, ID 1-389 and 392-396 were used. Conjugation was confirmed via R-phycoerythrin-labelled anti-rabbit (111-116-144 , RRID: AB_2337985) and anti-mouse IgG antibody (115-116-146, RRID: AB_2338629) (Jackson ImmunoResearch Laboratories). Pooled SBAs were stored at 4°C.

**Antibody scoring scheme**

Antibodies were selected based on a confidence scoring scheme (Table S3). Internal control beads (empty bead, rabbit IgG, anti-albumin and anti-human IgG) received a penalty score of -1. Antibodies correlating with any of the control beads (Spearman’s rho, ρ ≥ 0.4) were given score 0. The remaining antibodies were included in comparative analyses. Correlation was calculated for each antibody; in repeated assays (intra-assay correlation), between other antibodies sharing the protein target (paired antibody correlation) and between three independent, orthogonal methods: (i) a sandwich immunoassay developed for the plasma secretome,^15^ (ii) multiplex proximity extension assay (Olink Bioscience, Uppsala Sweden)^16^ and (iii) MS for targeted proteomics.^17^ If ρ < 0.4, then no score was added. For antibodies with ρ ≥ 0.4, a given score (2, 3 or 4 depending on comparison) plus the added mean or maximal correlation value (ρ_mean_ or ρ_max_). For correlations between orthogonal methods, one outlier subject (W0030) was excluded from the analysis. Antibodies that were not included in the orthogonal methods but did have a cis-pQTL hit were given an additional score of 4. In the case of plate variability that remained after batch normalization, predominantly seen in antibodies with high MFI signal due to signal saturation, a penalty score (-log_10_(P-value)/10) derived from a Kruskal-Wall rank sum test) was subtracted.

The final confidence score of an antibody consisted of the sum of scores calculated from all correlations. Antibodies that did not receive any score from the aforementioned criteria were set as failed with a total score of 1. Antibodies with score >1 passed the initial filtering. Lastly, one antibody profile per protein target was chosen by selecting the highest scoring binder. In the case that two antibodies with the same intended target also shared the same score, the mean signal intensity was calculated and used in downstream analysis (Table S4). Two cis-pQTL hits for CFH were included as unique protein targets. In total, 734 features were selected.

**Orthogonal proteomics assays for antibody validation**

***Sandwich immunoassays***

An extensive bead-array based sandwich immunoassay (SIA) was performed for screening and validating ~1,800 antibody pairs against 209 protein targets. Coupling of capture antibodies to MagPlex beads (Luminex Corp.) and sample incubation was prepared according to the protocol detailed in the SBA method. Biotinylation of detection antibodies was performed as previously described.^18^ Briefly, 2 µg of each antibody diluted in PBS-T was incubated with 5 µl protein A-coated magnetic beads (30 mg/ml, Dynabeads, 10002D, Invitrogen), washed and biotinylated with a 150x molar excess of EZ-Link-NHS-PEG4-Biotin (21329, Thermo Scientific) dissolved in DMSO (276855, Sigma-Aldrich). The labeled detection antibodies were washed and dissociated from the beads with 0.2 M acetate (97064-482, VWR), pH 3.2 (elution buffer). Next, 0.5 M Tris-base (T6066, Sigma-Aldrich), pH 8 was added to buffer the solution. Finally, PBS-T was added to obtain a final antibody concentration of 0.072 µg/µl. Biotinylated antibodies were stored at 4°C. The performance of each antibody capture-detection pair was assessed in dilution series of recombinant protein standards and in a human mixed gender plasma pool. Next, protein targets overlapping with the single-binder Wellness SBA were measured with SIA in samples collected from 18 Wellness participants, including samples from all four visits. For protein quantification, the measured MFI signals were converted to concentration using the dilution curves of the protein standard with a 5-parametric fit and multiplying it with the sample dilution factor.

***Proximity extension assay data***

A total of 978 unique plasma proteins were measured in 101 Wellness participants using a multiplexed proximity extension assay.^16^ Eleven panels were included: Cardiometabolic, Cell Regulation, Cardiovascular II and III, Development, Immune Response, Immuno-Oncology, Oncology II, Inflammation, Metabolism, Neurology, and Organ Damage. Each panel consist of a microtiter plate measuring 92 proteins in 90 samples, including 96 pairs of antibody probes tagged with DNA reporter molecules. In brief, 1 µl of each sample was incubated with 3 µl of probe solution and incubated overnight at 4°C. Next, 96 µl extension solution containing enzyme and PCR reagents was added. Signal intensities were quantified using a BioMark HD System (Fluidigm Corp.). Data was normalized using an internal control (extension control) and an external control (added to the plate in triplicate), and then transformed using a pre-determined correction factor. The resulting data were provided using the arbitrary unit Normalized Protein eXpression (NPX) on a log2 scale, which were linearized by using the formula 2NPX. The limit of detection for each measured protein was set to three standard deviations above the background.

***Mass spectrometry***

Plasma samples were first diluted 10 times in 100 mM Tris (pH 8) buffer and 5 µl of diluted sample corresponding to 0.5 µl plasma was mixed with QPrEST (Table S7) mastermix that was prepared on the Bravo liquid handler to represent a 1:1 (L:H) peptide ratio with the endogenous levels in plasma if possible. Plasma dilution and QPrEST addition were done using reverse pipetting technique. Proteins were first denatured with 1% sodium deoxycholate (SDC) and reduced with 10 mM dithiothreitol (DTT) for 10 min at 90 °C and alkylated by addition of 50 mM chloroacetamide (CAA) and incubated in dark for 20 min. A mixture of Pierce MS grade porcine trypsin (Thermo Scientific) and Lys-C (Wako) was added manually in respective enzyme to substrate ratios (1:50 for trypsin and 1:100 for Lys-C). After 16 h of incubation at 37 °C the digestion was quenched by adding TFA to a final concentration of 0.5% (v/v). SDC was precipitated for 30 min at RT and then centrifuged for 10 min at 3,273 rcf on Allegra X 12R centrifuge (Beckman-Coulter, Brea, CA, USA). The sample was cleaned by using in-house prepared StageTips packed with Empore C18 Bonded Silica matrix (3M, Saint Paul, MN, USA. Briefly, three layers of octadecyl membrane were placed in 200 μl pipette tips. The membrane was activated by addition of 100% ACN and then equilibrated with 0.1% TFA. Then, 15 µg of peptides from the acidified sample (half of the sample digestion volume) was added to the StageTip membrane and then washed twice with 0.1% TFA. The peptides were eluted in two-step elution with 30 µl of elution buffer (80% ACN, 0.1% FA) in each step. In between every buffer addition during the desalting the samples were centrifuged for 3 min at 931 rcf. Desalted peptides were vacuum‐dried and stored at -20 °C before subjected for LC‐MS/MS analysis.

In total, 432 samples were measured using UltiMate 3000 binary RS nano-liquid chromatography system (Thermo Fisher) with an EASY-Spray ion source connected to an on-line Q Exactive HF mass spectrometer (Thermo Fisher). Samples were resuspended by the autosampler prior LC-MS/MS analysis. Briefly, 1 µg peptide weight was loaded onto an Acclaim PepMap 100 trap column (75 μm × 2 cm, C18, 3 μm, 100 Å, Thermo Scientific), washed with 100% of solvent A (3% ACN, 97% H2O, 0.1% FA), and then separated by PepMap RSLC C18 column (75 µm x 25 cm, 2 µm, 100 Å, Thermo Scientific). Peptides were eluted with a linear gradient of solvent B (95% ACN, 5% H20, 0.1% FA). The column was kept at 35°C by the in-source temperature controller. Protein quantification was performed according to a previously established Parallel Reaction Monitoring (PRM) method (Supplementary Data file S2). Full MS scans were acquired at 60,000 resolution (AGC target 3e6, mass range 350-1,600 m/z and injection time 110 ms), followed by 20 MS/MS scans at 30,000 resolution (AGC target 2e5, NCE 27, isolation window 1.5 m/z and injection time 55 ms) which were defined by a scheduled (2 min windows) PRM isolation list that contained 174 paired light and heavy peptide precursors (n(peptides) = 87) from 55 QPrESTs directed towards 52 human proteins. The raw MS files from the study samples were processed in Skyline (version 3.7) and analysed in R (version 3.4.1).

**Supplementary Statistical Analysis**

**Processing of data with AbsPQN**

We developed the antibody specific probabilistic quotient normalization (AbsPQN), a data processing strategy for antibody SBAs that is based on probabilistic quotient normalization (PQN).^19^ PQN generates a sample specific correction factor to account for differences in intensity levels between samples. However, PQN applies the same correction for all antibodies within a data set, hence, a more antibody specific normalization factor is needed. Our aim with AbsPQN was to extract the unique feature of each antibody and to minimize the influence of measured background signal.

First, the PQN normalization was applied as described by Dieterle et al., on a data matrix where rows correspond to samples and columns are antibodies

1. The median of each antibody was calculated
2. The data matrix with raw data (MFI_raw_) was divided by the antibody median vector, creating a scaled data matrix (MFI_scaled_)
3. The median of each sample was calculated from MFI_scaled_, creating the PQN denominator (pqnD) vector
4. MFI_raw_ was divided by pqnD to create a PQN normalized data matrix (MFI_PQN_)

The correlation between each antibody and pqnD was obtained and one antibody specific factor (ASF) per antibody was calculated

1. ASF_i_ = (cor(pqnD, MFI_i_) ☓ (pqnD – 1)) + 1

where MFI_i_ is the median fluorescence intensity and ASF_i_ is the antibody specific normalization factor for the ith antibody.

AbsPQN normalized values was obtained by dividing the PQN normalized data matrix by a vector containing all ASF_i_

1. MFI_AbsPQN_ = MFI_PQN_ ÷ ASF

Following AbsPQN, batch effects arising from the analysis with several plates were minimized by the multi-MA normalization approach.^20^ Using the AbsPQN normalization, the correlation between random antibody pairs was reduced and the correlation to negative controls diminished (Fig. S12). The AbsPQN data correlated with unadjusted MFI intensity levels, preserved correlations in replicated assays, and maintained validated correlations of paired antibodies.

**Analysis of seasonal variation**

For the seasonal association analysis, a model for regular cyclic movements across time was fitted to each protein profile. In order to take a possible phase shift into account, both the sine and the cosine terms were included in the linear regression model, based on the following axiom of trigonometry

$$cos(\theta-\alpha)=cos(\theta)cos(\alpha)+ sin(\theta)sin(\alpha)$$

$$=cos(\alpha)cos(\theta)+ sin(\alpha)sin(\theta)$$

$$=\beta_{1}\cos\left( \theta\right)+ \beta_{2}sin(\theta)$$

where α is the phase shift, β_1_ = cos(α) and β_2_ = sin(α).

Hence,

$$y_{i}=\beta_{1}\cos\left( t \right)+ \beta_{2}+ t+ \varepsilon$$

where y*_i_* is the measured signal intensity for the *i*th antibody*, t* is the sampling time expressed in radians, and *ε* is the random error component.

Including both the cosine and the sine in the linear regression, β1 and β2 was estimated by the model. The phase shift α was computed

$$\alpha=arctan(\beta2/\beta1)$$

The significance of the effect of season was examined by the fitted model

$$\hat{y}=\hat{\beta_{1}}\cos\left( t- arctan\left( \frac{\beta_{2}}{\beta_{1}} \right) \right) + t$$

For each variable we observed the fit to the model (P-value), the phase shift (α), and the date at the highest peak, i.e. at the amplitude (the estimate).

**Weighted Correlation Network Analysis**

***Data preprocessing and filtering***

WGCNA^21,22^ was performed both on the full set of antibodies and on the filtered set of 734 protein features, as described in the Supplement. For both the full set and the selected set of proteins the data was log transformed prior to analysis. The outliers were removed by visual inspection of dendrograms of individuals for each visit separately. Only one outlier (W0030) was identified across all visits. The same individual was also identified and removed in correlation analyses.

***Defining protein modules***

Protein modules were defined using the WGCNA v. 1.66^21,22^ in R v. 3.5.1. Hyperparameters were estimated as recommended by the authors. The minimum module size was set to 10. Modules of proteins were defined and modules with eigengene Pearson correlation > 0.75 were merged into mega modules.

***Within-visit module stability***

The within-visit module stability was assessed using a bootstrapping approach. We created 50 bootstrap samples each consisting of a random selection of 90% of the subjects. WGCNA was then performed for each bootstrap for each visit. The module membership of each protein across the bootstrap samples can then be compared to assess whether the protein is consistently assigned to a specific module or not within each visit.

***Defining longitudinally stable core patterns of mega modules***

Overlap of modules were considered in a sequential manner (i.e. comparing visits 1 and 2,
2 and 3, and 3 and 4). Significant overlaps (P > 0·05) between mega modules were computed using Fisher’s exact test/hypergeometric test for the overlap of two modules.^23^ All significant sequential overlaps between mega modules across visits were used to determine core patterns of overlap. Proteins following a core pattern across all visits were assigned to that core pattern.

***Pathway enrichment analysis***

We performed pathway enrichment using the online tool ConsensusPathDB,^24^ which incorporates a range of pathway databases. We here performed over-representation analysis using hypergeometric testing on each pattern and using the full filtered set of proteins as the background. Results from ConsensusPathDB were then summarized to assign the biological functions to each core pattern with the most evidence across several pathway databases (Supplementary Data file S4).

***Associations to clinical traits***

Association to clinical traits and antibodies was tested for per visit by performing a linear regression model for continuous variables, and analysis of variance (ANOVA) for categorical variables. P-values were controlled for multiple correction by FDR. Associations that were significant (FDR P ≤ 0·05) in all four visits were included in unsupervised clustering. For continuous variables the correlation between antibodies and variables were determined by Spearman’s rho. For categorical variables the proportion of variance was determined by Eta squared.

In the WGCNA analysis, each core pattern was associated to clinical traits using the eigengene of the proteins included in the pattern. Eigengenes were defined as the first principal component of the proteins in the pattern. Eigengenes were computed for each visit separately and were then associated to clinical traits, which were also measured at each visit. Thus, each association between a core pattern and a clinical trait was calculated four times. This both serves as a replication and an assessment of the stability of the association over the course of one year.

**Annotation of facets of individual and longitudinal protein profiles**

Protein data was standardized to z-scores and a linear model was applied for every individual per protein (four measurements per participant). Three variables; intercept, slope, and sum of residuals were extracted for each individual and protein, and the variables were compared to the population mean.

Protein and individual-specific cut-offs were set for each of the three variables

$$\bar{x}_{i}\pm3 ☓ {SD}_{i}$$

where *x̄* is the mean across participants, SD is the standard deviation across participants and *i* is the *i*th protein. Hence, a personalized profile was built that described if an individual is within or above/below the assigned cut-off. Individuals with an intercept greater or smaller than the mean intercept indicated an elevated or decreased baseline compared to the population mean. Slopes outside of the cut-off captured individuals that have a gradual increase or decrease in protein level through time. The sum of residuals captured increased or decreased protein abundance at specific timepoints. Individual protein profiles were further studied by combining the information of intercept (“baseline”), slope (“trend”) and sum of residuals (“fluctuation”) per protein. Each measurement within the cut-off range received score 0, while measurements outside of the cut-off received score 1 (Supplementary Data file S4). The accumulated score for each individual was calculated by taking the sum of protein scores. The distribution of profiles of all subjects except one (W0030) were included in the analysis.

**Supplementary Figures**

Fig. S1. Distribution of antibody scores. In the initial set of 1,450 antibodies, each antibody received a score based on comparative analysis within the SBAs, other proteomics-based methods and pQTL analysis. The scoring system is weighted, giving an accumulative score based on (in increasing order): reproducibility, concordant protein profiles across proteomics methods, and proteins with *cis*-pQTL. Each dot represents one antibody bead. Antibodies selected for downstream statistical analysis are coloured green, control beads are marked in yellow, and excluded antibodies are marked in grey. SBA, suspension bead array.


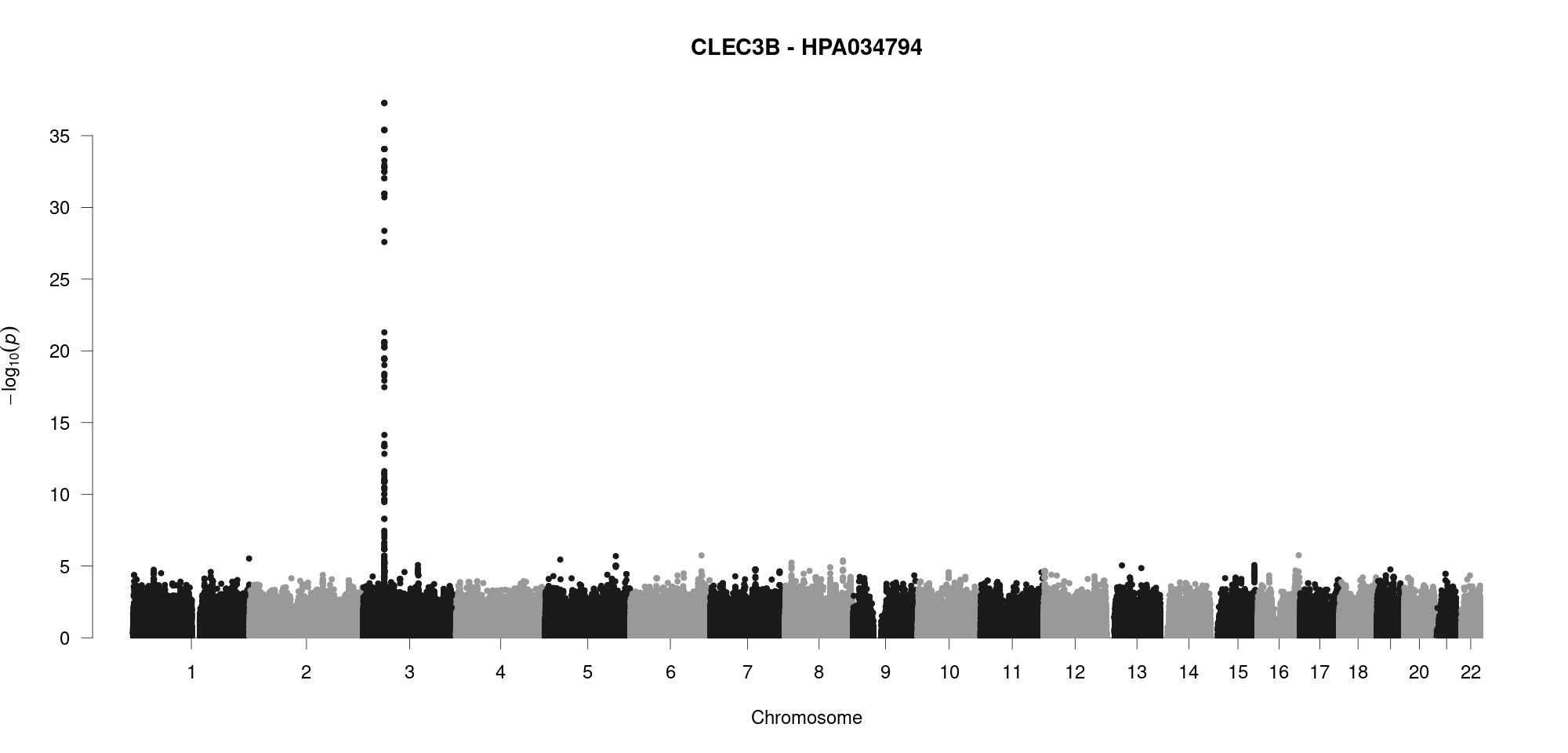


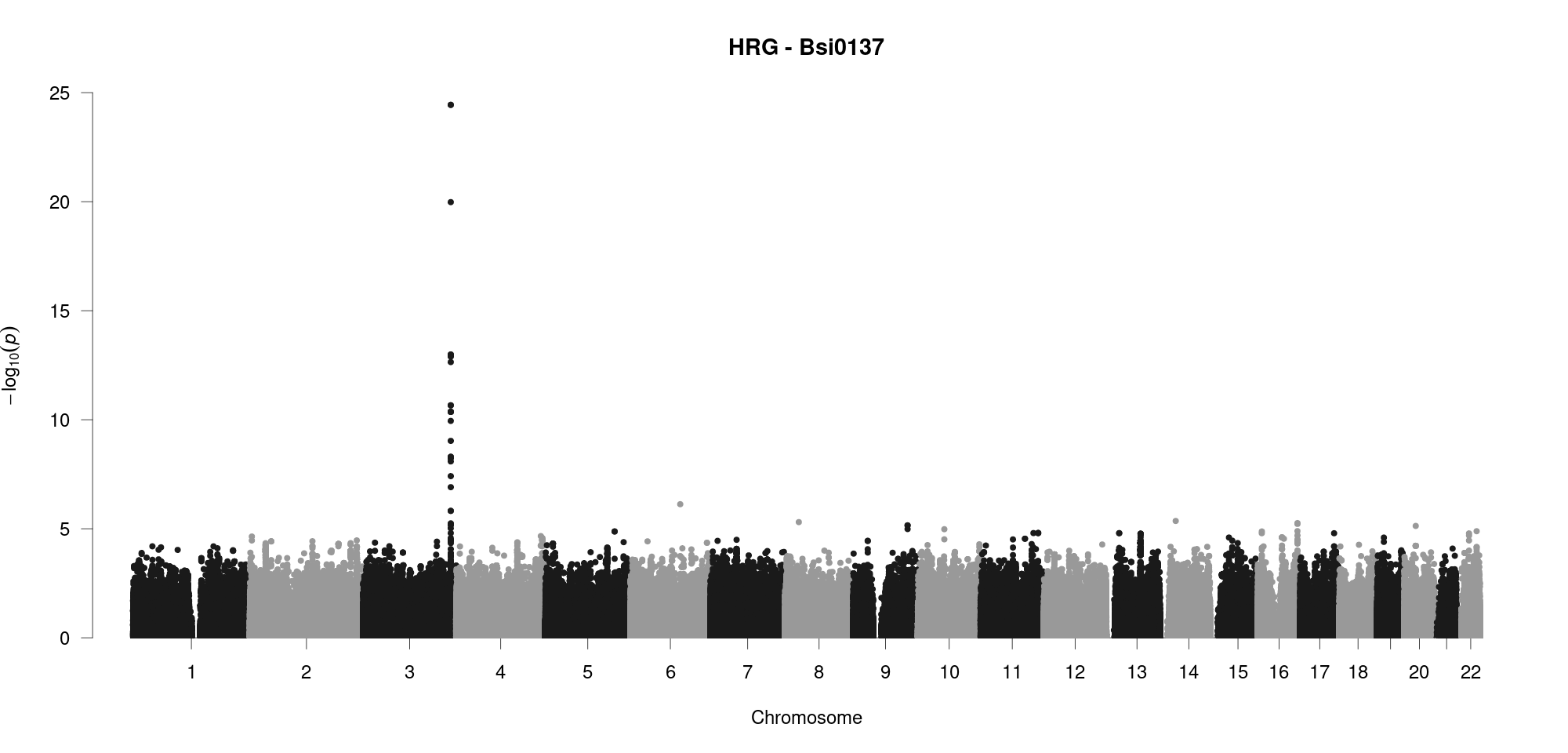


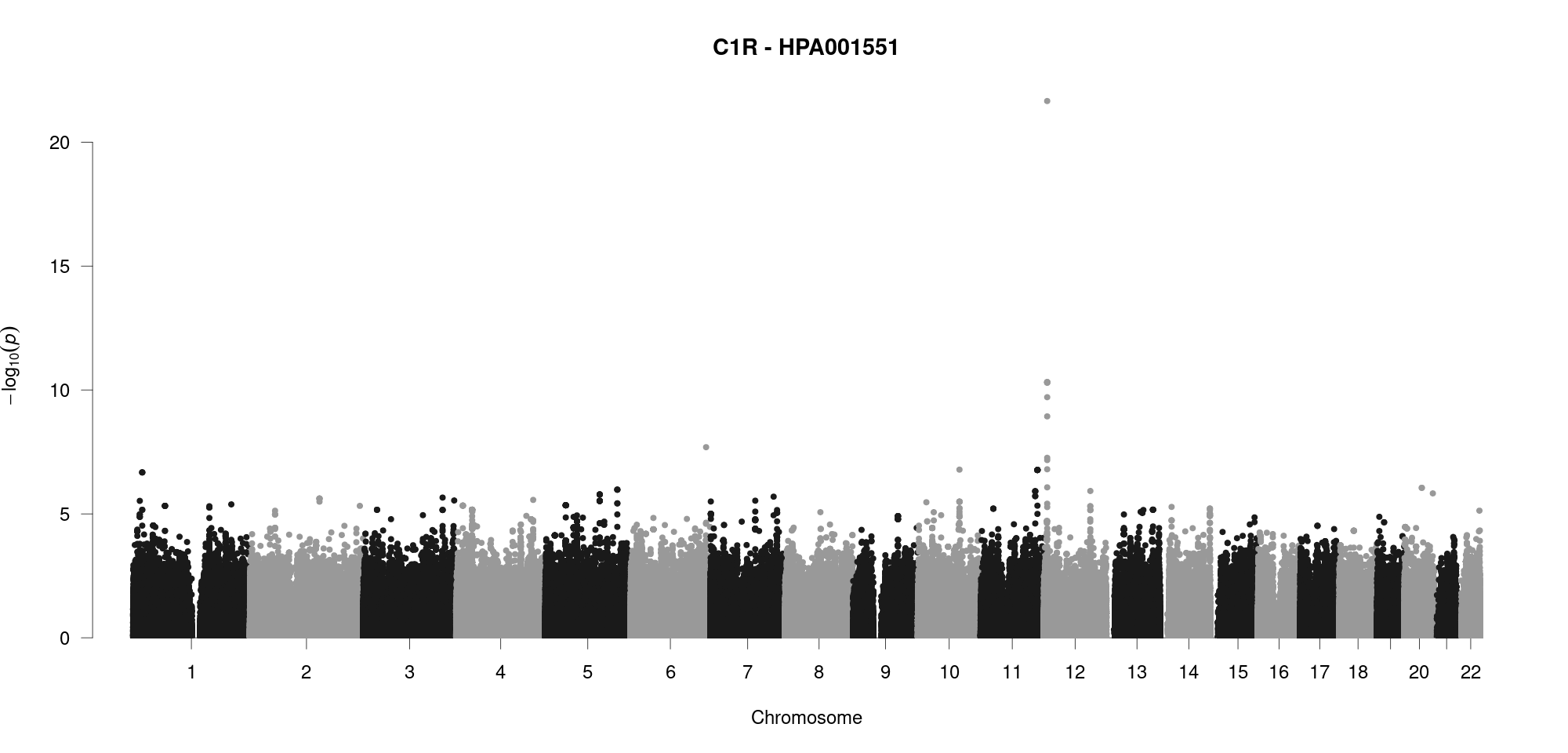


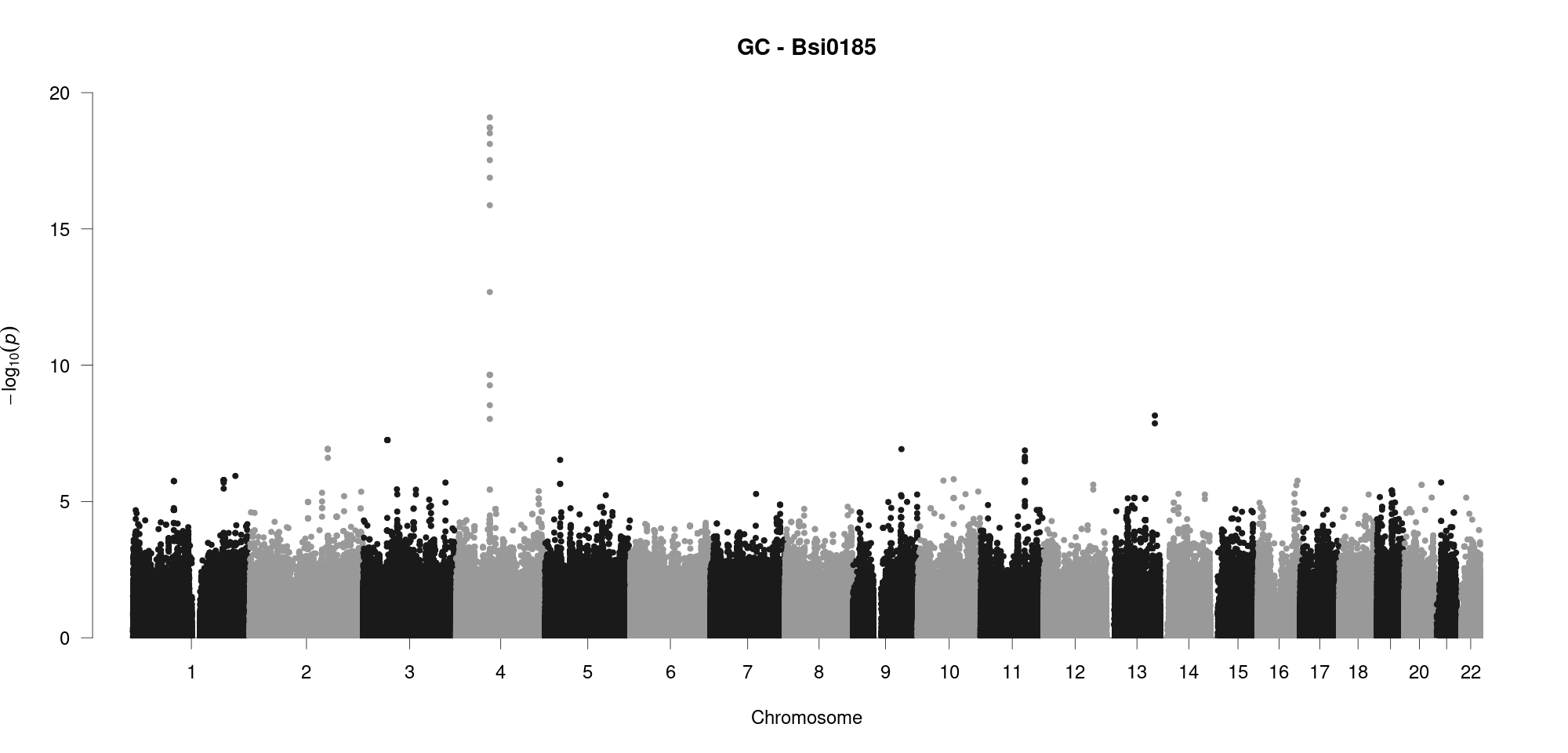


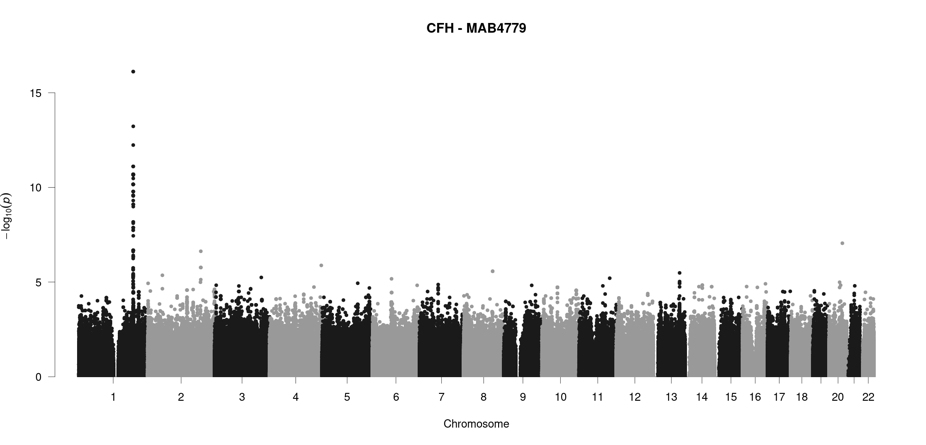


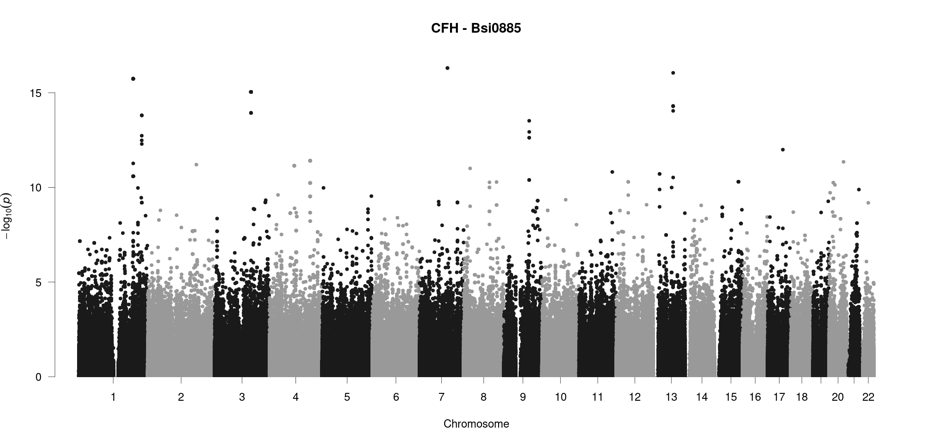


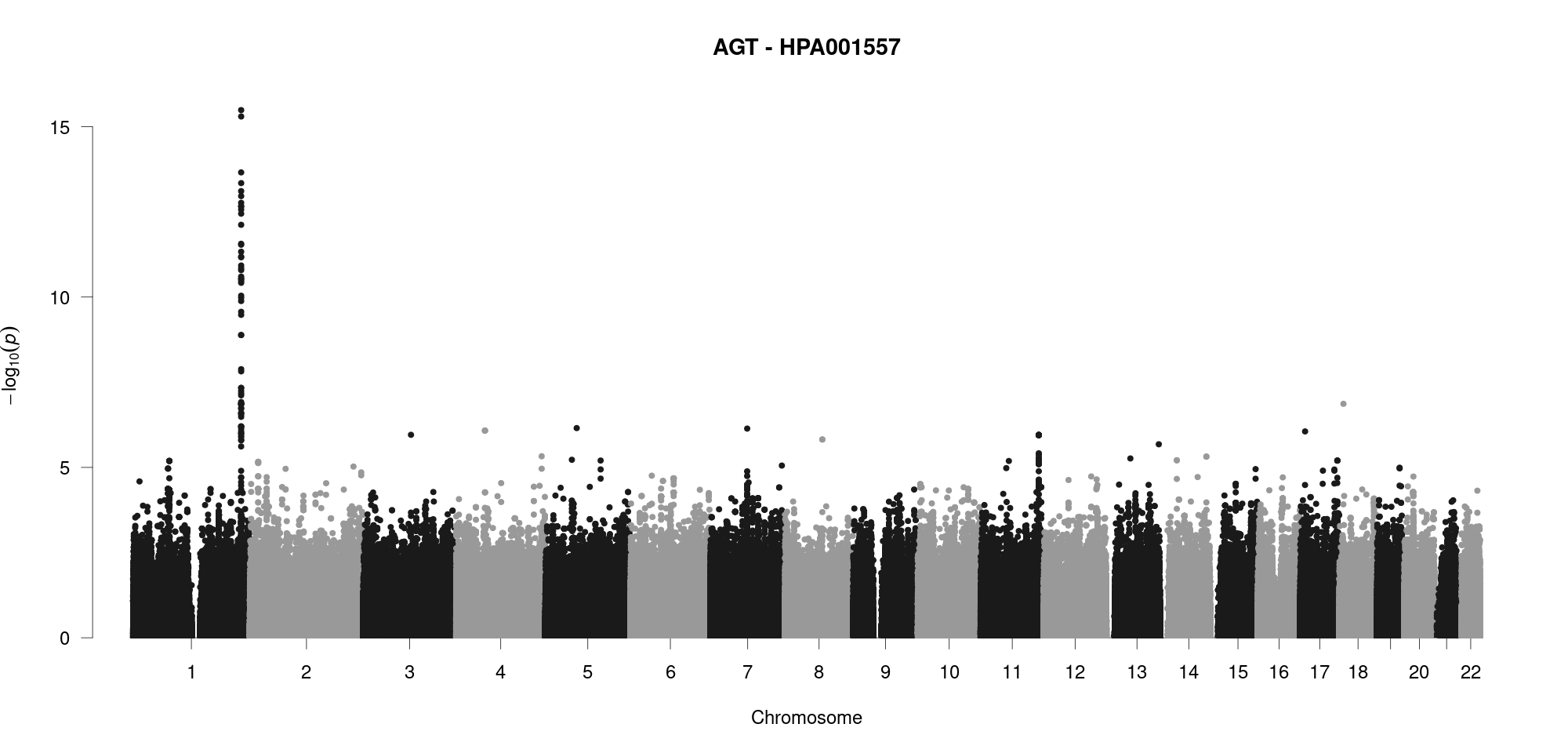


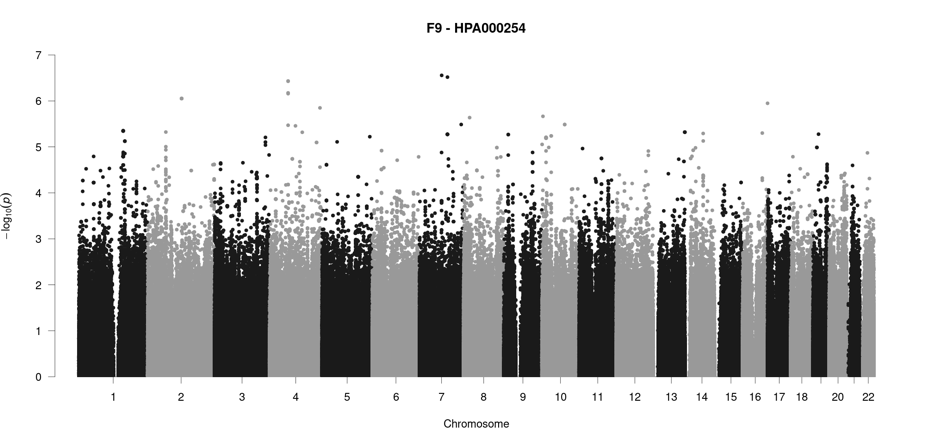


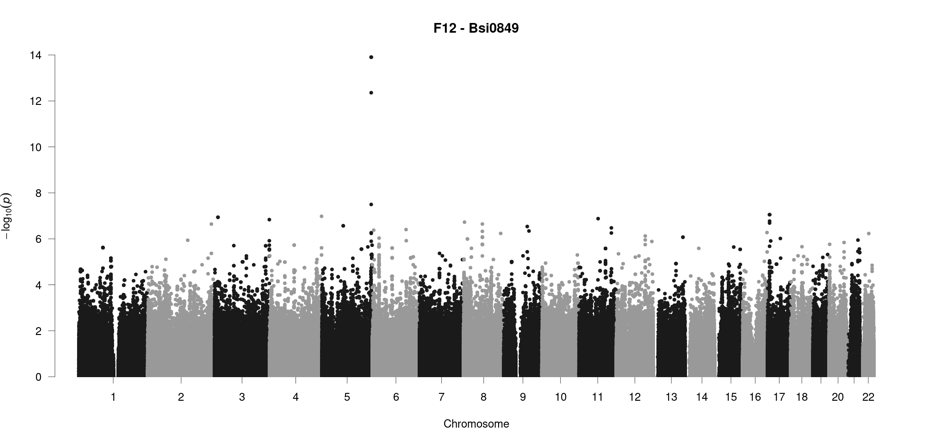


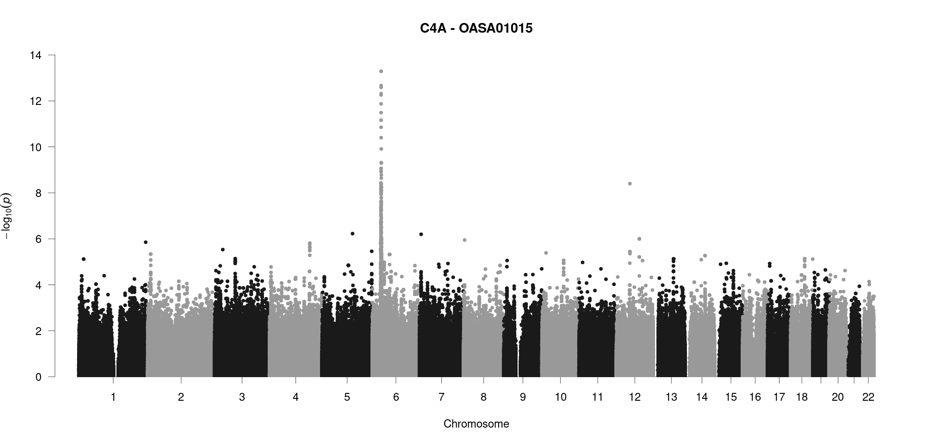


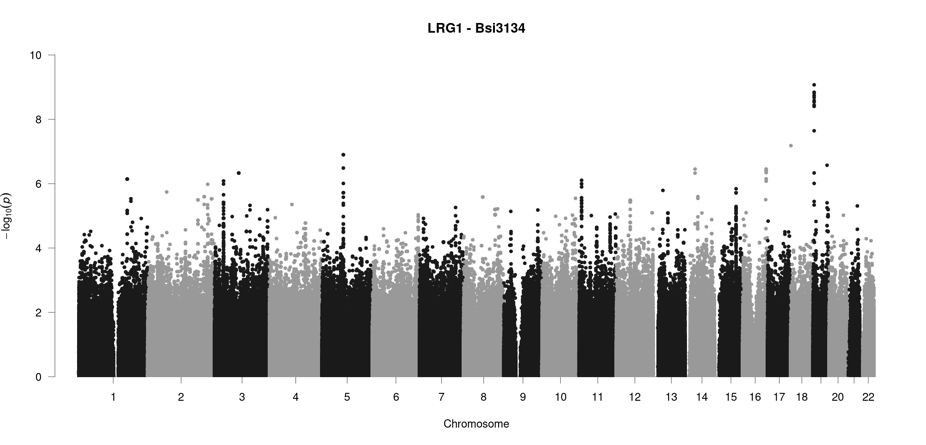


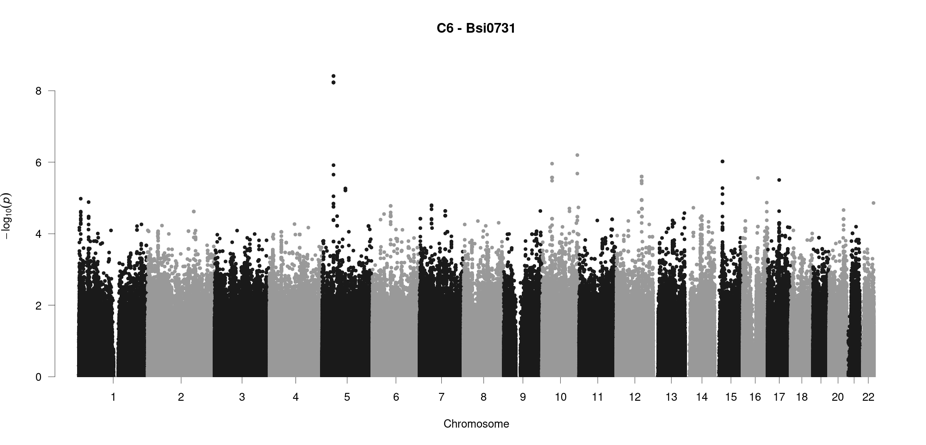


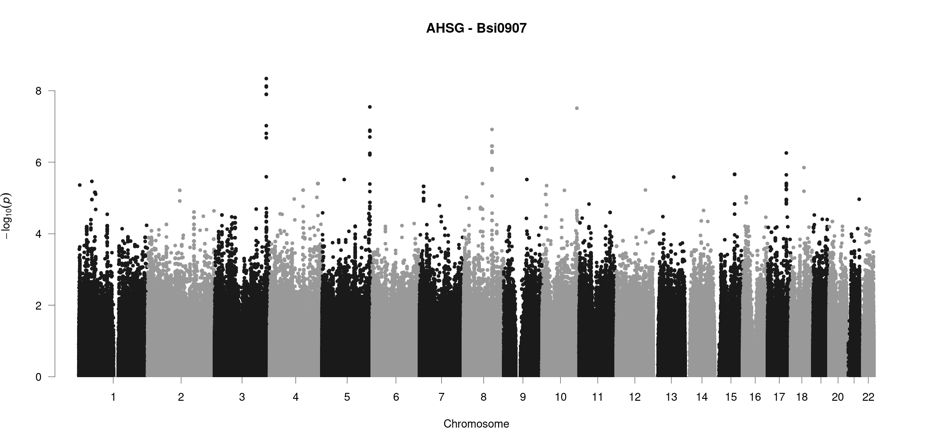


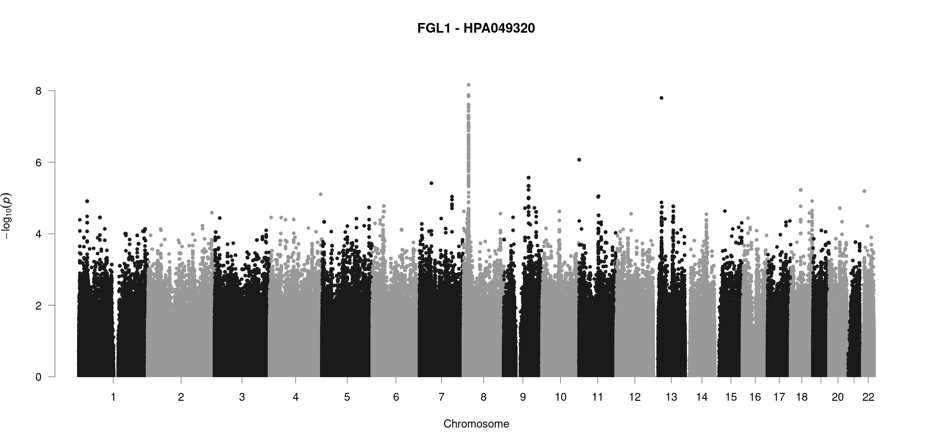


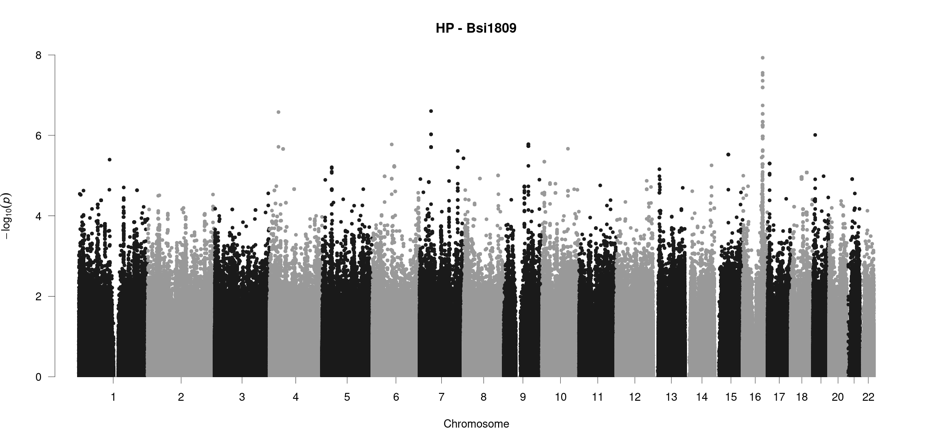


Fig. S2. Manhattan plots. Manhattan plots of 15 proteins with significant *cis*-pQTLs. Chromosomal positions are shown along the x-axis, while the y-axis reports -log_10_(P-value) from linear regression models. The highest points in each plot are the SNPs more strongly associated with the protein profiles.

Fig. S3. Count of statistically significant P-values from clinical association test. Distribution of FDR P-values from linear mixed models. X-axis denotes clinical traits, y-axis corresponds to the number of significantly associated proteins (FDR P < 0·01).


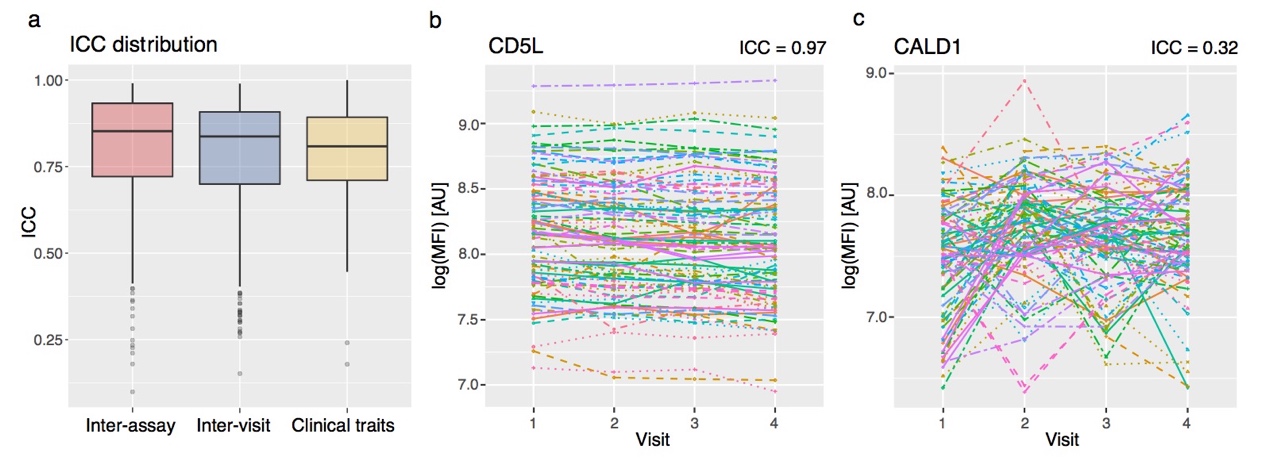


Fig. S4. Assessment of longitudinal protein profiles. (A) Distribution of protein ICC levels measured by SBA; inter-assay (red) and inter-visit (blue) correlations, and ICC distribution of clinical traits (yellow). (B) Protein CD5L levels and (C) CALD1 measured at consecutive visits. Each dot represents one individual at one visit occasion and is connected with a line to the following visit measurement. ICC, intraclass correlation; MFI, median fluorescence intensity; AU, arbitrary unit.


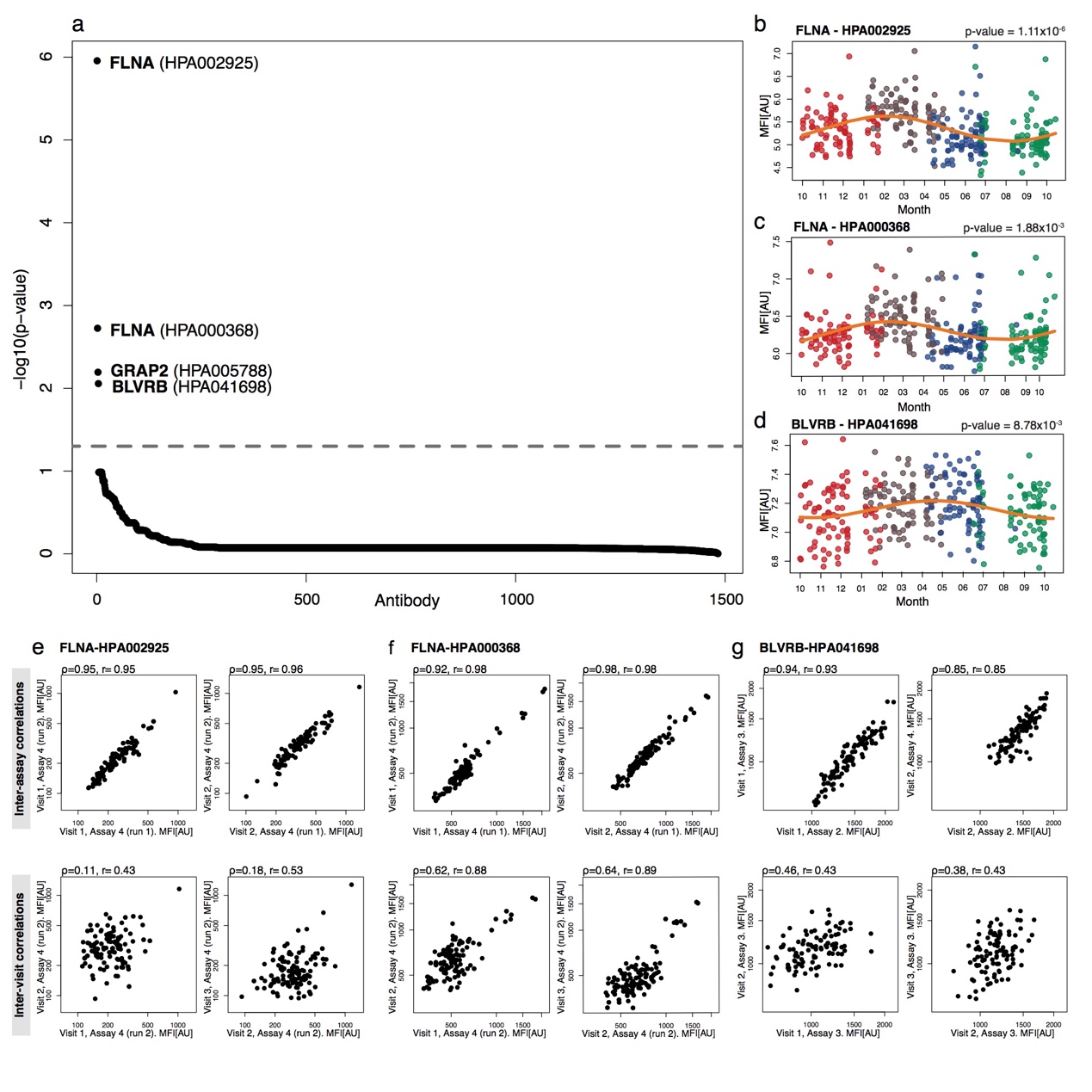


Fig. S5. Seasonal variation in protein profiles. (A) Distribution of FDR P-values from seasonal association model. Global results from association test with measured protein and seasonal fluctuations. Each dot represents the -log_10_(P-value) of one antibody. Significant associations are labelled. Horizontal line = P-value at 0.05. (B)-(D) Seasonal variation across one year in (B-C) Filamin-A (FLNA) and (B) Biliverdin reductase B (BLVRB). Y-axis shows time in months and x-axis signal intensity. Each dot represents one individual, colour correspond to visit occasion. Orange line corresponds to model fit.
(E)-(G) Inter-assay correlations (upper panel) and inter -visit correlations for (E)-(F) FLNA and (G) BLVRB. MFI, median fluorescence intensity; AU, arbitrary unit. Correlations are indicated by ρ (Spearman’s Rho) and r (Pearson correlation coefficient).

Fig. S6. Inter-assay and inter-visit variability per sample. Shown are correlations for SBA1-2 of technical (inter-assay, upper panel in blue) and longitudinal (inter-visit, lower panel in green) profiles. (A) subject W0086 and (B) subject W0065. Each dot represents one protein from SBA1 and SBA2. MFI relates to median fluorescent intensity and AU are arbitrary units. Correlations are indicated by ρ (Spearman’s Rho) and r (Pearson correlation coefficient).SBA, suspension bead array.

Fig. S7. Dendrogram of proteins and their module membership in each visit. Dendrograms from hierarchical clustering used as the basis for the WGCNA clustering. The coloured barcodes at the bottom of each plot indicates the module membership of each protein. The top barcode shows the WGCNA modules obtained from the full data set, which are the modules used for downstream analyses. The remaining barcodes represent the WGCNA results from the bootstrap samples. This illustrates the overall consistency of the modules resulting from WGCNA within each visit. Colours are assigned according to the size of each module which may vary between bootstrap samples. Therefore, though colour assignment may vary between samples, module membership within each visit remains relatively stable across bootstraps.

Fig. S8. Matching of overlap between modules from sequential visits. Every module in one visit are compared to all modules in the next visit. Each cell contains the number of proteins common to the two modules being compared and the colour indicates the -log_10_(P-value) of the comparison computed by Fisher’s exact test. Each individual module is annotated by the number of proteins contained.

Fig. S9. Venn diagrams of overlap between mega modules across visits. Each circle represents one visit and contains all proteins assigned to the given mega module in that visit. Only proteins that follow the pattern across all four visits are included in the core set of proteins for that pattern. Patterns that contain no proteins are not shown.

Fig. S10. Plasma protein levels in TwinGene. Measured levels of cis-pQTL hits (A) Haptoglobin (HP), (B) Factor H (CFH), (C) Vitamin D binding protein (GC) and (D) Coagulation factor XII (F12) in TwinGene (n = 2,974). Each dot represents one individual. Signal intensities are standardized to z-scores.


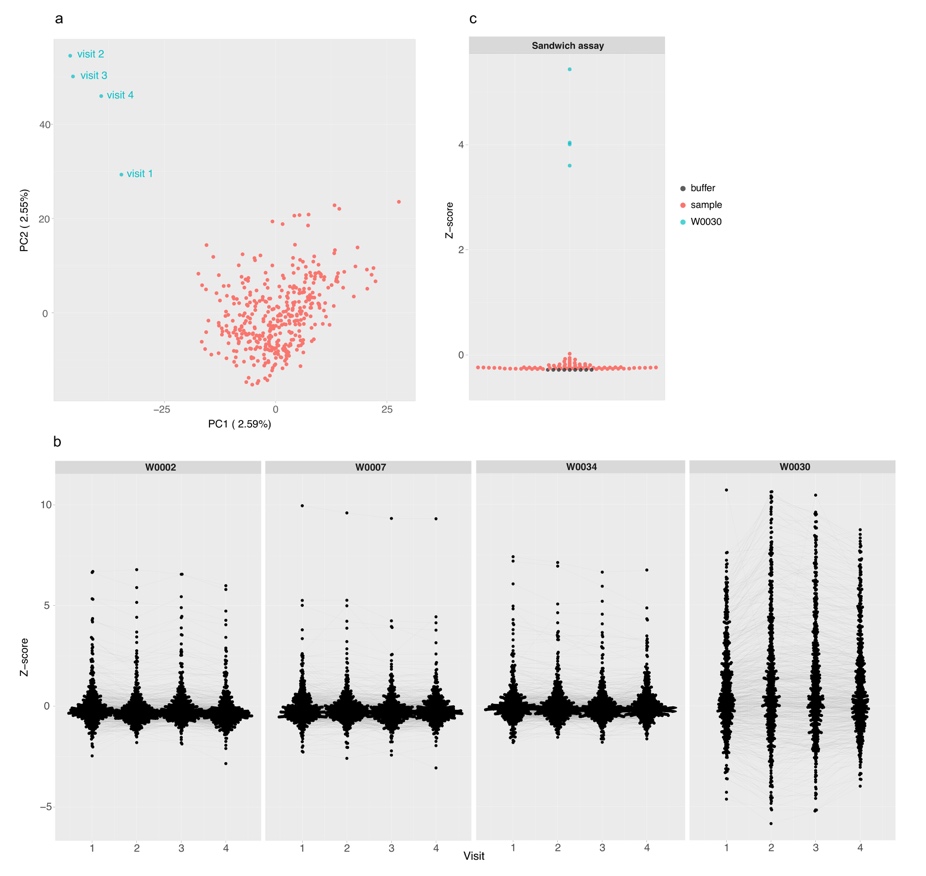


Fig. S11. Identification of IgM interference outlier. (A) Principal component analysis of samples from all four visits that have passed quality control; here 101 subjects and 734 protein features. (B) Protein profiles per visit of three randomly selected participants (W0002, W0007 and W0034) and the outlier W0030. X-axis denotes visit, y-axis displays z-scored signal intensities. (C) IgM sandwich assay measuring elevated IgM levels. Here, samples from all four visits from the outlier and 18 additional subjects are included. Turquoise dots correspond to the individual with high levels of IgM (W0030), remaining subjects are coloured red and buffer samples are coloured dark grey. Black dots represent one protein profile at one visit occasion and is connected with a line to the following visit measurement.

Fig. S12. SBA data normalization with AbsPQN. (A) Distribution of antibody correlations to control beads. Left, MA normalized data; right, AbsPQN data. (B) Correlation between paired antibodies sharing the same target protein versus any randomly selected pairs of antibodies exemplified by visit 3 and 4. Left, MA normalized data; right, AbsPQN data.

Table S1. Demographic characteristics in S3WP. Data on subject characteristics of all 101 participants. For categorical variables the number (n) of cases are given, and for continuous numerical data the mean(SD) and median(IQR) are displayed. All measurements are at baseline (visit 1). SD, standard deviation; IQR, interquartile range.

| **Category** | **Abbreviation** | **No. of cases** | **Mean**±**SD** | **Median(IQR)** |
| --- | --- | --- | --- | --- |
| Number of individuals (women/men) |  | 53/48 |  |  |
| Age at first visit (years) |  |  | 56.9±4.1 | 56(6) |
| Anthropometry |  |  |  |  |
| Body weight (kg) |  |  | 76.7±14.2 | 77.8(22.1) |
| Height (cm) |  |  | 173.7±10.6 | 173(16) |
| Body mass index (kg/m2) |  |  | 25.3±3.8 | 25.1(5.3) |
| Waist circumference (cm) |  |  | 94.3±10.4 | 95(15) |
| Hip (cm) |  |  | 102.7±6.8 | 102(8) |
| Body fat content (%) | Fat |  | 26.1±8.1 | 26.3(13.1) |
| Body bone content (%) | Bone |  | 3.7±0.4 | 3.7(0.6) |
| Body muscle content (%) | Muscle |  | 70.2±7.7 | 69.9(12.5) |
| Systolic blood pressure (mmHg) | SBP |  | 120.8±16.8 | 120(21) |
| Diastolic blood pressure (mmHg) | DBP |  | 81.1±9.8 | 80(13) |
| Place of birth |  |  |  |  |
| Sweden |  | 84 |  |  |
| Other European countries |  | 11 |  |  |
| Non-European countries |  | 6 |  |  |
| Smoking |  |  |  |  |
| Current smoker (yes/no) |  | 3/98 |  |  |

**Table S2.** Clinical measurements in S3WP. Clinical traits for all 101 participants. For categorical variables the number (n) of cases are given, and for continuous numerical data the mean(SD) and median(IQR) are displayed. All measurements are at baseline (visit 1). SD, standard deviation; IQR, interquartile range.

| **Measurement** | **Abbreviation** | **Mean**±**SD** | **Median(IQR)** |
| --- | --- | --- | --- |
| Glucose homeostasis |  |  |  |
| Glucose (mmol/L) | Gluc | 5.5±0.5 | 5.4(0.7) |
| Hemoglobin A1c fraction (mmol/mol) | HbA1c | 33.7±3.1 | 34(4) |
| Blood lipids |  |  |  |
| Total cholesterol (mmol/L) | Chol | 5.8±0.9 | 5.8(1.3) |
| Low-density lipoprotein cholesterol (mmol/L) | LDL | 3.7±0.9 | 3.7(1) |
| High-density lipoprotein cholesterol (mmol/L) | HDL | 1.9±0.6 | 1.8(0.8) |
| Triglycerides (mmol/L) | TG | 1.1±0.6 | 0.9(0.7) |
| Apolipoprotein A1 (g/L) | ApoA1 | 1.8±0.3 | 1.8(0.3) |
| Apolipoprotein B (g/L) | ApoB | 1.1±0.3 | 1.0(0.3) |
| Fraction Apolipoprotein B/Apolipoprotein A1 | ApoB/apoA1 | 0.7±0.2 | 0.6(0.2) |
| Liver markers |  |  |  |
| Alanine aminotransferase (µkat/L) | ALAT | 0.4±0.2 | 0.4(0.2) |
| Gamma-glutamyltransferase (µkat/L) | GGT | 0.4±0.4 | 0.3(0.2) |
| Kidney markers |  |  |  |
| Creatinine (µmol/L) | Crea | 78.2±13.9 | 76(21) |
| Cystatin C (mg/L) | CystC | 0.9±0.1 | 0.9(0.1) |
| Heart markers |  |  |  |
| N-terminal pro b-type natriuretic peptide (NT-pro-BNP) (ng/L) | NTproBNP | 67.1±47.8 | 56(47) |
| Troponin T, high sensitivity (ng/L) | TNT | 6.2±2.7 | 5.0(1.3) |
| Erythrocytes |  |  |  |
| Red blood cell count (x10*12/L) | RBC | 4.7±0.4 | 4.7(0.5) |
| Hemoglobin (g/L) | Hb | 141.6±11.6 | 144(17) |
| Hematocrit (erythrocyte volume fraction) (L/L) | Hct | 0.4±0.03 | 0.4(0.05) |
| Mean corpuscular volume (fL) | MCV | 91.2±4.9 | 92(6) |
| Mean corpuscular hemoglobin (pg) | MCH | 30.4±1.7 | 30(3) |
| Mean corpuscular hemoglobin concentration (g/L) | MCHC | 333.1±9.9 | 334(14) |
| Thrombocytes |  |  |  |
| Platelet count (x10*9/L) | Plt | 246.9±53.4 | 241(74) |
| Leukocytes |  |  |  |
| White blood cell count (x10*9/L) | WBC | 5.3±1.3 | 5.1(1.6) |
| Neutrophil count (x10*9/L) | Neut | 2.9±1.0 | 2.8(1.1) |
| Lymphocyte count (x10*9/L) | Lymph | 1.9±0.5 | 1.8(0.5) |
| Monocyte count (x10*9/L) | Mono | 0.3±0.1 | 0.3(0.1) |
| Eosinophil count (x10*9/L) | Eos | 0.2±0.1 | 0.1(0.1) |
| Basophil count (x10*9/L) | Baso | 0.01±0.04 | 0.0(0.0) |
| Other clinical chemistry variables |  |  |  |
| C-reactive protein, high sensitivity (mg/L) | CRP | 1.4±1.9 | 0.8(1.0) |
| Urate (µmol/L) | Urate | 297.8±67.1 | 284(79) |
| 1,25 Dihydroxy Vitamin D (pmol/L) | VitD | 128.9±45.8 | 131(69) |

Table S3. Antibody scoring scheme. Assessment criteria of antibodies and given scores. Ab, antibody; ρ, Spearman’s rho; ρ_mean_, mean correlation; ρ_max_, highest correlation between paired correlations.

|  | **Description of category** | **Score** | **Supportive** | **Not supportive** | **Not in category** |
| --- | --- | --- | --- | --- | --- |
| 1 | Internal control beads (penalty) | -1 | 33 | 1450 | - |
| 2 | Ab-control correlation* | 0 | 264 | 1219 | - |
| 3.1 | Inter-assay Ab correlation* | 2 + ρ_mean_ | 1305 | 178 | - |
| 3.2 | Paired Ab correlation* | 3 + ρ_max_ | 221 | 787 | 475 |
| 3.3 | Orthogonal method correlation* | 4 + ρ_max_ | 68 | 566 | 849 |
| 3.4 | GWAS (*cis*-pQTL) | 4 | 15 | 1435 | 33 |
| 3.5 | Plate effect variability ** (penalty) | -(-log_10_(P-value)/10) | 432 | 1051 | - |
| 4 | Failed Ab but not internal control | 1 | 138 |  | 1345 |
| 5 | Supportive Abs | > 1 | 1131 | 319 | - |
|  |  |  |  |  |  |

* Correlation cut-off ρ ≥ 0.4; ** P-value from Kruskal-Wallis rank sum test between microtiter plates.

**Table S4.** Selection outcome for antibodies and genes. Number of antibodies selected in downstream analysis and the corresponding number of unique target genes. Abs, antibodies.

| **Description of category** | **Abs (N)** | | **Genes (N)** | |
| --- | --- | --- | --- | --- |
|  | **Assessed** | **Not in category** | **Assessed** | **Not in category** |
| All Abs | 1483 | - | 876 | - |
| Paired Abs (excluding controls) | 1008 | 475 | 425 | 451 |
| Supportive Abs (score > 1) | 1131 | 385 | 733 | 143 |
| Combined Abs with same target* | 38 | 1093 | 18 | 715 |
| Selected Abs** | 734 | 749 | 733 | 143 |
|  |  |  |  |  |

* Select among Abs with same target the Ab with highest scores or combine data if score is concordant.
** Two Abs with *cis*-pQTLs for CFH were included.

**Table S5.** Annotation of targets of selected 734 protein features. The targets used for phenotypic description of the participants were annotated based on the destination of secretion (blood, other) or leakage. The table also shows the genes classified by the blood cell type specificity of their RNA expression (consensus distribution) (9). Corresponding gene names for the 734 protein features were annotated with Ensembl v. 92.38 (10), resulting in 751 unique gene names.

|  | Blood cell type expression specificity | | | | |  |
| --- | --- | --- | --- | --- | --- | --- |
|  | All | Many | Some | Single | Not detected | Total |
| Secreted to blood | 7 | 54 | 68 | 35 | 69 | 233 |
| Secreted to other tissue | 1 | 6 | 25 | 14 | 31 | 77 |
| Leakage | 13 | 11 | 15 | 6 | 10 | 55 |
| Not detected | 130 | 125 | 51 | 35 | 45 | 386 |
| Total | 151 | 196 | 159 | 90 | 155 | 751 |

Table S6. Protein profiles with cis-pQTLs (continued).

| Protein | Antibody† | P^‡^ | Top SNP^‡^ | Location^‡^ | Non-synon LD; proxy* | R^2^ | eQTL (Yes/No) | eQTL main tissue/cells | sQTLs (Yes/No) | sQTL main tissue |
| --- | --- | --- | --- | --- | --- | --- | --- | --- | --- | --- |
| CLEC3B | HPA034794 | 5.31 × 10^-38^ | rs4683026 | Intron | rs13963 : Gly - Ser | 1 | Y | Nerve - Tibial | Y | Adipose - Visceral (Omentum) |
| HRG | Bsi0137 | 3.65 × 10^-25^ | rs12493926 | Intron | rs1042464 : Asn - Ile | 1 | N |  | N |  |
| C1R | HPA001551 | 2.18 × 10^-22^ | rs1801046 | Exon | itself : Leu - Ser | 1 | N |  | N |  |
| GC | Bsi0185 | 8.13 × 10^-20^ | rs843005 | Intron | rs7041 : Asp - Glu | 1 | N |  | N |  |
| CFH | MAB4779 | 7.60 × 10^-17^ | rs61818923 | 99kb dn |  |  | Y | Nerve - Tibial | Y | Spleen |
| CFH | Bsi0885 | 1.81 × 10^-16^ | rs1048663 | Intron | rs1065489 : Glu - Asp | 1 | Y | Cultured fibroblasts | Y | Liver |
| AGT | HPA001557 | 3.26 × 10^-16^ | rs4762 | Exon | itself : Thr - Met | 1 | Y | Skin - Sun Exposed | N |  |
| F9 | HPA000254 | 5.16 × 10^-16^ | rs422187 | Intron | rs6048 : Thr - Ala | 1 | N |  | N |  |
| F12 | Bsi0849 | 1.25 × 10^-14^ | rs1801020 | 5' UTR |  |  | Y | Liver | N |  |
| C4A | OASA01015 | 5.15 × 10^-14^ | rs386480 | 3.0kb up |  |  | Y | Thyroid | Y | Thyroid |
| LRG1 | Bsi3134 | 8.50 × 10^-10^ | rs10426311 | 5.2kb up |  |  | Y | Liver | N |  |
| C6 | Bsi0731 | 3.88 × 10^-9^ | rs7443604 | Intron | rs1801033 : Ala - Glu | 1 | Y | Artery - tibial | Y | Nerve - Tibial |
| AHSG | Bsi0907 | 4.58 × 10^-9^ | rs13073106 | 3.0kb dn | rs4918 : Ser - Thr | 1 | N |  | Y | Liver |
| FGL1 | HPA049320 | 6.83 × 10^-9^ | rs10093134 | Intron |  |  | Y | Pancreas | N |  |
| HP | Bsi1809 | 1.18 × 10^-8^ | rs811053 | 156kb dn |  |  | N |  | Y | Adipose - Visceral (Omentum) |

*The R^2^ is the coefficient of determination between the top SNP and the corresponding LD proxy

Table S7. Spike in levels of QPrESTs.

| PrEST | Gene | Spike in [fmol/0.5 ul of plasma] based on BCA* | Spike in [fmol/0.5 ul of plasma] based on LC-MS | Off-ratio on purpose |
| --- | --- | --- | --- | --- |
| HPRR670085 | AFM | 480 | 447.323 | 1 |
| HPRR670086 | AFM | 485 | 288.714 | 1 |
| HPRR350062 | AGT | 600 | 890.786 | 1 |
| HPRR350074 | AHSG | 820 | 831.46 | 5 |
| HPRR2760276 | ALB | 4201 | 2516.065 | 100 |
| HPRR350094 | AMBP | 1289 | 801.908 | 2 |
| HPRR3450265 | APOA1 | 2510 | 2812.475 | 10 |
| HPRR260124 | APOA4 | 375 | 290.917 | 1 |
| HPRR3720311 | APOB | 825 | 958.962 | 1 |
| HPRR2760373 | APOD | 1000 | 608.995 | 4 |
| HPRR350088 | APOL1 | 350 | 259.917 | 1 |
| HPRR280083 | C1QC | 800 | 227.454 | 1 |
| HPRR280085 | C1S | 350 | 258.867 | 1 |
| HPRR280086 | C1S | 300 | 293.42 | 1 |
| HPRR280090 | C3 | 1050 | 799.978 | 2 |
| HPRR4060158 | C4BPB | 175 | 137.946 | 1 |
| HPRR2550054 | C8B | 450 | 249.774 | 1 |
| HPRR3720238 | CD5L | 500 | 512.905 | 1 |
| HPRR2760280 | CLEC3B | 300 | 293.304 | 1 |
| HPRR650017 | CPB2 | 90 | 121.97 | 1 |
| HPRR350053 | CPN2 | 800 | 312.714 | 1 |
| HPRR4190255 | ECM1 | 53 | 63.344 | 1 |
| HPRR350069 | F13B | 165 | 54.069 | 1 |
| HPRR3790030 | F2 | 1165 | 970.418 | 1 |
| HPRR3790031 | F2 | 1065 | 1005 | 1 |
| HPRR350051 | F5 | 15 | 80.579 | 1 |
| HPRR4220446 | FGG | 2700 | 3150.204 | 10 |
| HPRR232446 | FLNA | 150 | 137.321 | 1 |
| HPRR3760281 | GSN | 455 | 592.801 | 1 |
| HPRR4200150 | HPX | 600 | 442.724 | 5 |
| HPRR3730089 | HRG | 800 | 1636.908 | 1 |
| HPRR380014 | IGF2 | 525 | 120.089 | 1 |
| HPRR3140236 | IGFALS | 90 | 234.101 | 1 |
| HPRR350077 | IGFBP3 | 225 | 122.502 | 1 |
| HPRR350060 | IGJ | 1120 | 728.819 | 1 |
| HPRR3140086 | ITIH1 | 650 | 601.255 | 1 |
| HPRR350008 | KLKB1 | 250 | 137.914 | 1 |
| HPRR350146 | KNG1 | 931 | 139.73 | 2 |
| HPRR2350028 | KRT1 | 70 | 33.449 | 1 |
| HPRR350016 | LRG1 | 950 | 761.993 | 1 |
| HPRR3280117 | PGLYRP2 | 340 | 323.711 | 1 |
| HPRR2320064 | PLG | 700 | 666.156 | 1 |
| HPRR1510029 | PPBP | 700 | 594.144 | 1 |
| HPRR330194 | PROC | 37 | 21.492 | 1 |
| HPRR4190038 | RBP4 | 1200 | 421.607 | 1 |
| HPRR320021 | SERPINA1 | 2000 | 3357.225 | 10 |
| HPRR140864 | SERPINA10 | 37 | 35.975 | 1 |
| HPRR320016 | SERPINA3 | 920 | 673.72 | 5 |
| HPRR140840 | SERPINA6 | 600 | 555.988 | 1 |
| HPRR330198 | SERPINC1 | 1250 | 1100.079 | 2 |
| HPRR350052 | SERPINF2 | 725 | 883.028 | 1 |
| HPRR670072 | TFRC | 15 | 3.297 | 1 |
| HPRR350065 | THBS1 | 100 | 115.253 | 1 |
| HPRR1310071 | TLN1 | 20 | 41.903 | 1 |
| HPRR4030673 | VTN | 1350 | 1013.519 | 1 |

*Colorimetric protein concentration assay using bovine serum albumin standard. BCA, bicinchoninic acid assay.

Data file S1 (separate file). Antibody overview. Table of affinity reagents, antibody providers, RRID, antibody scores and selected antibody features with IQR.

Data file S2 (separate file). PRM method details. Details on protein quantification by PRM method.

Data file S3 (separate file). Functional annotation of core patterns. Results from over representation analysis performed with ConsensusPathDB on 2019.12.19.

Data file S4 (separate file). Protein and clinical associations using linear mixed effects models. Table of variables and FDR P-values as reported in Fig. 2 and Fig. S3.

Data file S5 (separate file). Facets of individual and longitudinal protein profiles. Table of scores for each individual and target measurement, and summary scores per individual and protein target.

References

1. Hoofnagle AN, Wener MH. The fundamental flaws of immunoassays and potential solutions using tandem mass spectrometry. *J Immunol Methods* 2009; **347**(1-2): 3-11.

2. Nguyen TT, Baumgarth N. Natural IgM and the Development of B Cell-Mediated Autoimmune Diseases. *Crit Rev Immunol* 2016; **36**(2): 163-77.

3. Paludo J, Ansell SM. Advances in the understanding of IgM monoclonal gammopathy of undetermined significance. *F1000Res* 2017; **6**: 2142.

4. Rajkumar SV, Dimopoulos MA, Palumbo A, et al. International Myeloma Working Group updated criteria for the diagnosis of multiple myeloma. *Lancet Oncol* 2014; **15**(12): e538-48.

5. Hu J, Lu J, Goyal A, et al. Opposing FlnA and FlnB interactions regulate RhoA activation in guiding dynamic actin stress fiber formation and cell spreading. *Hum Mol Genet* 2017; **26**(7): 1294-304.

6. Wu S, Li Z, Gnatenko DV, et al. BLVRB redox mutation defines heme degradation in a metabolic pathway of enhanced thrombopoiesis in humans. *Blood* 2016; **128**(5): 699-709.

7. Ljubica Perisic Matic, Maria Jesus Iglesias, Mattias Vesterlund, et al. Biliverdin Reductase B as a Marker of Intraplaque Hemorrhage. *JACC: Basic to Translational Science* 2018; **3**(4): 464-80.

8. Dopico XC, Evangelou M, Ferreira RC, et al. Widespread seasonal gene expression reveals annual differences in human immunity and physiology. *Nat Commun* 2015; **6**: 7000.

9. Stranneheim H, Engvall M, Naess K, et al. Rapid pulsed whole genome sequencing for comprehensive acute diagnostics of inborn errors of metabolism. *Bmc Genomics* 2014; **15**.

10. Green RC, Berg JS, Grody WW, et al. ACMG recommendations for reporting of incidental findings in clinical exome and genome sequencing. *Genet Med* 2013; **15**(7): 565-74.

11. Machiela MJ, Chanock SJ. LDlink: a web-based application for exploring population-specific haplotype structure and linking correlated alleles of possible functional variants. *Bioinformatics* 2015; **31**(21): 3555-7.

12. Magnusson PK, Almqvist C, Rahman I, et al. The Swedish Twin Registry: establishment of a biobank and other recent developments. *Twin Res Hum Genet* 2013; **16**(1): 317-29.

13. Lichtenstein P, De Faire U, Floderus B, Svartengren M, Svedberg P, Pedersen NL. The Swedish Twin Registry: a unique resource for clinical, epidemiological and genetic studies. *Journal of Internal Medicine* 2002; **252**(3): 184-205.

14. Hong M-G, Dodig-Crnković T, Chen X, et al. Levels of histidine-rich glycoprotein variants in human blood are associated to chronological age and predict mortality. *bioRxiv* 2019.

15. Häussler RS, Bendes A, Iglesias M, et al. Systematic Development of Sandwich Immunoassays for the Plasma Secretome. *Proteomics* 2019; **19**(15): e1900008.

16. Assarsson E, Lundberg M, Holmquist G, et al. Homogenous 96-plex PEA immunoassay exhibiting high sensitivity, specificity, and excellent scalability. *PloS One* 2014; **9**(4).

17. Edfors F, Forsstrom B, Vunk H, et al. Screening a Resource of Recombinant Protein Fragments for Targeted Proteomics. *J Proteome Res* 2019; **18**(7): 2706-18.

18. Dezfouli M, Vickovic S, Iglesias MJ, Nilsson P, Schwenk JM, Ahmadian A. Magnetic bead assisted labeling of antibodies at nanogram scale. *Proteomics* 2014; **14**(1): 14-8.

19. Dieterle F, Ross A, Schlotterbeck G, Senn H. Probabilistic quotient normalization as robust method to account for dilution of complex biological mixtures. Application in H-1 NMR metabonomics. *Anal Chem* 2006; **78**(13): 4281-90.

20. Hong MG, Lee W, Nilsson P, Pawitan Y, Schwenk JM. Multidimensional Normalization to Minimize Plate Effects of Suspension Bead Array Data. *J Proteome Res* 2016; **15**(10): 3473-80.

21. Langfelder P, Zhang B, Horvath S. Defining clusters from a hierarchical cluster tree: the Dynamic Tree Cut package for R. *Bioinformatics* 2008; **24**(5): 719-20.

22. Zhang B, Horvath S. A general framework for weighted gene co-expression network analysis. *Stat Appl Genet Mo B* 2005; **4**.

23. Langfelder P, Luo R, Oldham MC, Horvath S. Is My Network Module Preserved and Reproducible? *Plos Comput Biol* 2011; **7**(1).

24. Kamburov A, Pentchev K, Galicka H, Wierling C, Lehrach H, Herwig R. ConsensusPathDB: toward a more complete picture of cell biology. *Nucleic Acids Res* 2011; **39**(Database issue): D712-7.
